# Artificial intelligence in early warning systems for infectious disease surveillance: a systematic review

**DOI:** 10.3389/fpubh.2025.1609615

**Published:** 2025-06-23

**Authors:** Ismael Villanueva-Miranda, Guanghua Xiao, Yang Xie

**Affiliations:** ^1^Department of Health Data Science and Biostatistics, University of Texas Southwestern Medical Center, Dallas, TX, United States; ^2^Department of Bioinformatics, University of Texas Southwestern Medical Center, Dallas, TX, United States

**Keywords:** artificial intelligence, public health, disease surveillance, early warning system (EWS), infectious disease, systematic review

## Abstract

**Introduction:**

Infectious diseases pose a significant global health threat, exacerbated by factors like globalization and climate change. Artificial intelligence (AI) offers promising tools to enhance crucial early warning systems (EWS) for disease surveillance. This systematic review evaluates the current landscape of AI applications in EWS, identifying key techniques, data sources, benefits, and challenges.

**Methods:**

Following PRISMA guidelines, a systematic search of Semantic Scholar (2018-onward) was conducted. After screening 600 records and removing duplicates and non-relevant articles, the search yielded 67 relevant studies for review.

**Results:**

Key findings reveal the prevalent use of machine learning (ML), deep learning (DL), and natural language processing (NLP), which often integrate diverse data sources (e.g., epidemiological, web, climate, wastewater). The major benefits identified include earlier outbreak detection and improved prediction accuracy. However, significant challenges persist regarding data quality and bias, model transparency (the “black box” issue), system integration difficulties, and ethical considerations such as privacy and equity.

**Discussion:**

AI demonstrates considerable potential to strengthen infectious disease EWS. Realizing this potential, however, requires concerted efforts to address data limitations, enhance model explainability, ensure ethical implementation, improve infrastructure, and foster collaboration between AI developers and public health experts.

## 1 Introduction

Protecting global populations from the continuing threat of infectious diseases is an important concern in an increasingly interconnected world (1–3). Several factors increase the risk of pandemics, including the rising frequency of zoonotic spillovers (4), the growing challenge of antimicrobial resistance (5), widespread globalization (2), rapid urbanization (1), and the effects of climate change (6). These factors show the urgent need for strong surveillance and preparedness strategies (7, 8). In this context, the ability to rapidly detect and effectively respond to infectious disease outbreaks at their earliest stages is urgent (9, 10).

Artificial intelligence (AI) has emerged as a powerful tool in public health, offering new possibilities to improve infectious disease surveillance and early warning systems (1, 11, 12). Its potential to transform early outbreak detection, refine epidemiological models, and optimize healthcare responses has received growing attention (13–16). By using advanced algorithms to process and analyze large datasets from diverse sources, AI can identify patterns and detect anomalies that may signal emerging public health threats (1, 17–19).

This review critically examines the current state of AI applications in early warning systems (EWS) for infectious disease surveillance. It addresses the following key questions:

1. What are the primary artificial intelligence techniques and methodologies currently employed in early warning systems for infectious disease surveillance?
2. What types of data sources are predominantly utilized by these AI-driven systems?
3. What are the main reported benefits and advantages of applying AI in this domain?
4. What are the key limitations, challenges, and ethical considerations identified in the literature regarding the use of AI for infectious disease surveillance?
5. What are the emerging trends and future directions for the development and application of AI in this field?

## 2 Methods

This systematic review was conducted following the Preferred Reporting Items for Systematic Reviews and Meta-Analyses (PRISMA) guidelines (20) to ensure a transparent and repeatable process. The search strategy, detailed below, was specifically designed to retrieve studies relevant to the primary research questions presented in the Introduction.

### 2.1 Search strategy

The review began by defining four main search topics to capture the breadth of relevant literature: (1) the application of artificial intelligence in early warning systems for the detection and management of infectious diseases; (2) the use of machine learning techniques to enhance early warning systems for infectious disease surveillance; (3) the role of deep learning methods in developing early warning systems for infectious diseases; and (4) the use of AI-driven early warning systems for infectious disease outbreaks. These topics were chosen to cover the key technologies and applications in the field.

Search queries were generated based on these topics and executed on Semantic Scholar (the primary database used, *n* = 1 in the PRISMA diagram). For each topic, three types of queries were developed: a broad query, a focused query using the “+” operator, and a related query including additional supporting terms. This multi-strategy approach was designed to balance the retrieval of a wide range of studies with the identification of highly relevant results. Table 1 presents the full set of search queries.

**Table 1:** Search topics and query strategies for the systematic review.

**Search topic**	**Broad query**	**Focused query**	**Related query**
The application of artificial intelligence in early warning systems for the detection and management of infectious diseases.	“Artificial intelligence early warning systems infectious diseases”	“Artificial intelligence” + “early warning systems” + “Infectious diseases”	“Machine Learning” + “Predictive analytics” + “Epidemiology”
The use of machine learning techniques to enhance early warning systems for infectious disease surveillance.	“Machine learning early warning systems infectious diseases surveillance”	“Machine learning” + “Early warning systems” + “Infectious diseases surveillance”	“Artificial intelligence public health predictive analytics epidemiology”
The role of deep learning methods in developing early warning systems for infectious diseases.	“Deep learning early warning systems infectious diseases”	“Deep learning” + “Early warning systems” + “Infectious diseases”	“Machine learning” + “Predictive analytics” + “epidemiology”
The use of AI-driven early warning systems for infectious disease outbreaks.	“AI-driven early warning systems infectious diseases”	“AI-driven” + “Early warning systems” + “infectious diseases”	“Machine learning public health disease surveillance”

We selected Semantic Scholar as the primary data source for this review due to its extensive reach and advanced search functionalities. It is a free, AI-driven search engine indexing over 200 million academic papers, utilizing machine learning to identify relevant literature beyond simple keyword matching. Its foundation on comprehensive knowledge graphs, including the Microsoft Academic Knowledge Graph and Springer Nature's SciGraph, connected with direct partnerships with over 50 publishers and data providers, guarantees broad coverage of academic content (21).

### 2.2 Inclusion and exclusion criteria

To guarantee the relevance and focus of this review, specific inclusion and exclusion criteria were applied during the study selection process. Studies were included if they directly addressed one or more of the four research topics concerning AI, machine learning, deep learning, and early warning systems for human infectious disease surveillance.

Only journal articles, conference papers, or studies published in English from the year 2018 onward were included. Studies were excluded if they were identified as editorials, commentaries, or abstracts only; if they did not focus on human infectious diseases or early warning systems; or if they were not published in English. A total of 223 reports were excluded during the full-text assessment stage, primarily because they were not directly relevant to the research questions.

### 2.3 Study selection process

The initial search retrieved ~600 records. After removing 303 duplicate records during the identification phase, 297 unique records remained for screening.

**Figure 1:**
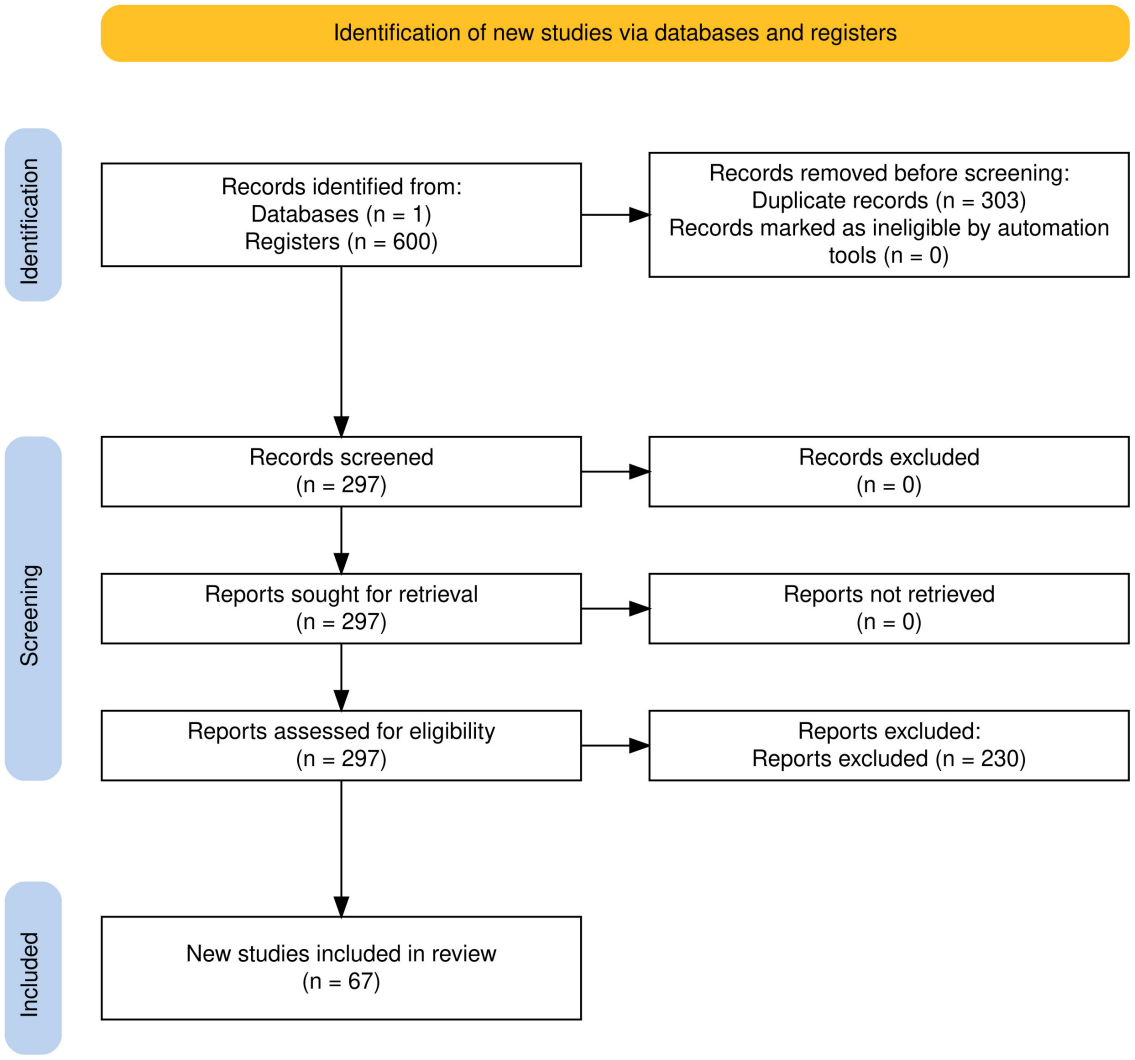
PRISMA flow diagram illustrating the selection process for studies included in the review.

All 297 records were screened based on their titles, abstracts. No records were excluded at this initial screening stage. The full texts of all 297 records were then retrieved and assessed for eligibility according to the inclusion and exclusion criteria described in Section 2.2. During the full-text assessment, 223 reports were excluded, mainly because they did not meet the relevance criteria. This process resulted in 67 studies being included in the final review, as shown in the PRISMA flow diagram (Figure 1).

## 3 Results

Following the systematic search and screening process detailed in the Methods section, a final set of 67 studies met the inclusion criteria and were included in this review (see PRISMA flow diagram, Figure 1). A summary of the key characteristics of the 67 included studies is presented in Table 2. This summary highlights aspects central to our research questions, including the primary AI/ML techniques employed (Research Question 1), the types of data sources used (Research Question 2), the main application areas of the AI systems, and the specific infectious diseases addressed.

**Table 2:** Characteristics of included studies.

**References**	**Technique(s) used**	**Data sources**	**Focus**	**Disease(s)**
Zhou et al. (4)	Systematic review, multi-trigger monitor system	Community level surveillance data	Summarize EID surveillance	Emerging infectious diseases (EIDs)
Li et al. (22)	Retrospective review, blockchain, smart contracts, sentiment analysis	WanFang Data, CNKI, WoS, PubMed, Health Depts, Hospitals, Social Media, Stats Bureau, Meteo Depts, wastewater	Review EWS progress	Infectious diseases
Sun (7)	–	Multi-source data	Enhance surveillance systems (China)	Infectious Diseases, EIDs
Yang et al. (13)	DL (SEAR Model), LR, SVM, RF, XGBoost, LSTM	ILI surveillance data (China)	Develop DL EWS model (SEAR)	Influenza
Zhang et al. (62)	–	Medical data (HIS, LIS, PACS, EMR)	Identify early warning signals in hospitals	Infectious diseases, EIDs
Fu et al. (59)	Light-controlled capillary NA separation, PCR, AI monitoring, IoT	POC testing device data	Develop sample-to-answer diagnosis platform	Emerging infectious diseases (EIDs)
Panah (1)	AI, Data analytics	Multi-source data	Introduce framework integrating AI with public health Sys	Infectious diseases
Zehnder et al. (58)	Hydraulic modeling, ML (SVM), FFT	Wastewater data (simulated), hydrodynamic models	Develop methodology for rapid pathogen source tracing	SARS-CoV-2
Javed et al. (29)	Systematic review (Kitchenham), ML, DL, Federated learning	IEEE, ACM, Springer, ScienceDirect databases	Review ID recognition & Propose federated learning framework	General infectious diseases
Meckawy et al. (23)	Systematic review, adapted CASP Checklist	PubMed, Scopus Databases	Review EWS effectiveness (outbreak detection)	Infectious diseases (Pandemic pot.)
Oeschger et al. (2)	AI, wastewater epidemiology, bioaerosol sampling, LFA, NA amplification	Climate data, health records, social media, sentinel animals, wastewater, Bioaerosols	Examine technologies for earlier ID detection	Emerging infectious diseases (EIDs)
Hu et al. (67)	–	–	Summarize EWS definitions, status, indicators	Infectious diseases
Tian et al. (28)	Blockchain, AI, big data, smart contracts	Multi-party monitoring data	Propose Blockchain EWS technology & framework	infectious diseases
Morin et al. (6)	Climate forecasting, exposure-response models	Temperature, precipitation, environmental data	Review use of climate/weather-driven EWS	Climate-sensitive IDs (Dengue, Cholera, RVF)
Bernasconi et al. (24)	Data-driven/knowledge-based analysis (AI implied)	Viral genomes	Develop methods for genomic surveillance (SENSIBLE)	Viral pathogens (COVID-19)
Hu et al. (68)	–	–	Summarize EWS definitions, status, models, methods	Infectious diseases
Ibiam et al. (44)	Systematic review (AI, ML, DL)	–	Review AI in clinical decision support	Infectious diseases (Sepsis)
Singh and Dhiman (17)	AI, predictive analytics (ML, DL, NLP, NN)	EHR, medical imaging, genetic data, wearables	Review AI predictive analytics for early detection	Multiple (cancer, CVD, diabetes, neonatal, IDs)
Raja and Sukanya (33)	AI, ML, DL, NLP	Real-time Data, IoT, social media analytics	Explore AI in public health surveillance	Infectious diseases (COVID-19)
Zhang et al. (38)	AI (deep learning)	Epidemic data (multi-source)	Develop AI real-time monitoring & response system	Infectious diseases
Srivastava et al. (3)	Review (AI, ML, DL, Image Recognition)	Patient data	review AI role in early diagnosis & Treatment	infectious diseases
Mckee et al. (14)	AI (predictive algorithms)	Social media, meteorological data, mobile data	Explain AI applications for pandemic management	Infectious diseases (pandemics)
Haval and Ikhar (54)	DL (CNN), SEM	Tuberculosis case statistics (city)	Develop CNN for early ID detection (CNN-IDD-PHE)	Tuberculosis (TB)
Nwankwo et al. (36)	AI, predictive analytics, ML	Health records, surveillance data, environmental	Discuss AI predictive analytics for rural epidemics	Epidemic diseases
Olaboye et al. (34)	AI, predictive models	Mobile health data (geolocation, apps, wearables)	Explore AI/mobile data for real-time surveillance	Infectious diseases
Isiaka et al. (15)	AI, ML, predictive modeling	EHR, social media, historical data, climate, mobility	Explore AI for early detection & management	infectious diseases (COVID-19)
Langford et al. (43)	Review (AI, ML, DL, LLMs)	–	Discuss AI disruption in ID workforce	Infectious diseases
Li et al. (11)	Review (AI, DL)	US COVID-19 mortality data (example)	Overview AI use in IDs (COVID-19 focus)	Infectious diseases (COVID-19)
Badidi (63)	Review (Edge AI, ML, DL, federated learning)	EHR, wearable devices, demographic information	Review edge AI for early health prediction	Chronic diseases, infectious diseases
Chu et al. (51)	AI, ML, DL	Medical imaging data (clinical & preclinical)	Assess AI techniques in ID imaging research	Infectious diseases (COVID-19)
Wong et l. (5)	Review (AI, ML, GNNs, Seq-to-Func/Struct, generative)	systems/synthetic biology data, drug screens	Discuss AI approaches for detecting/treating/understanding IDs	Infectious diseases (AMR)
Tran et al. (64)	Review (AI, ML, data fusion)	Host-response proteomic data	Overview AI/ML for ID diagnosis (immunocompromised)	Infectious diseases (sepsis, COVID-19, Fungal)
Parums (8)	Editorial (Mentions AI/ML, Genome Seq.)	–	Update on AI uses/limitations in surveillance	Infectious diseases (COVID-19)
Ekundayo (18)	ML (supervised—forecasting, unsupervised—clustering)	EHR, social media, climate data, genomic sequences	Predict outbreaks & enhance surveillance	Infectious diseases (influenza)
Jaswal et al. (35)	AI, ML	Patient data, EHRs	Develop predictive models for early warning signs	Chronic diseases (diabetes, HTN, CVD)
Cheng et al. (39)	Spatial autocorrelation, ML (ARIMA, ELM, SVR, Wavelet, RNN, LSTM), stacking (RBF/PSO)	COVID-19 case data (China), AIDS, PTB case data	Analyze spatial patterns & Predict ID trends	COVID-19, AIDS, pulmonary TB
Manshi (30)	ML (time-series forecasting, RF, LSTM)	Public health data (Epi, Demo, Env, weather, mobility, sentiment)	Forecast outbreaks using ML models	Influenza, dengue, COVID-19
Bandal (65)	ML, streamlit	datasets (general)	Predict multiple diseases using ML/streamlit	Chronic diseases, infectious diseases
Natrayan et al. (37)	ML (SVM, RF, K-means), data mining	–	Enhance ID surveillance & Outbreak management	Infectious diseases
Towfek and Elkanzi (25)	Review (AI, ML, DL, NN)	Genomic data, environmental data, patient info.	Review ML role in predicting ID spread	Infectious diseases (AMR, TB, Measles, COVID-19)
Mandepudi et al. (45)	AI, ML (SVM, LSTM), NLP	Medical datasets (symptoms, history, Tx plans)	Design AI medical chatbot for prediction/assistance	Infectious diseases
Setegn and Dejene (2025) (56)	ML (RF, bagging, GB, CatBoost, XGBoost, LGBM), XAI	clinical features dataset (GitHub)	Develop XAI for symptom-based detection	Monkeypox
Quigley et al. (2025) (66)	AI (EPIWATCH system)	Open source data (syndromic/specific outbreaks)	Assess EPIWATCH as surveillance tool	Respiratory illnesses, conflict zone IDs
Kashmar et al. (2025) (50)	Scoping review (ML models: BERT, AraBERT, GMDH-NN, LSTM, HMM)	Social media data, climate data, health data	Analyze use of social media data in AI-based EWS	Infectious diseases (COVID-19, Flu)
James et al. (2024) (16)	Predictive analytics (AI, ML)	Health records, environmental data, social determinants	Examine predictive analytics role in surveillance	diseases (general public health)
Eze et al. (2024) (19)	Review (AI, ML, NLP, predictive modeling)	Health data analytics	Explore AI analytics for early ID detection (US Strat.)	Infectious diseases
Abinaya et al. (2024) (47)	ML (SVM, KNN, LR, DT, RF, MLP)	Symptom/prognosis dataset (Kaggle)	Optimize VBD surveillance with ML classification	Vector-borne diseases (11 types)
Addaali et al. (2024) (41)	Review (ML, DL, XAI)	–	Highlight value of XAI in predicting/managing IDs	Infectious diseases (COVID-19)
Chen et al. (40)	ML (RF, SVM, etc.)	Nosocomial infection surveillance (NIS) data, hospital ops, drug use, temp.	Build risk assessment system using ML	Nosocomial infections
Morr et al. (9)	Systematic scoping review (AI, ML, DL, Ensemble)	–	Assess AI capability in epidemic/pandemic EWS	epidemics/pandemics
Zhang et al. (61)	Adaptive dynamic threshold method (ADTM), ML (supervised—MLSM, unsupervised—MLUM)	ILI statistics, Baidu Index, clinical data (Weifang)	Compare threshold vs. AI EWS approaches	Respiratory infectious diseases (flu, COVID-19)
Garcia-Vozmediano et al. (57)	ML (tree regression, RF, GB)	Food safety audits, human case data (Italy)	Develop ML EWS for foodborne outbreaks	*Salmonella*
Wattamwar et al. (2024) (55)	GIS, ML prediction models, time series	–	Present GIS-enabled real-time surveillance system	Lassa fever
El-kenawy et al. (26)	ML (linear regression)	Dengue cases, vector abundance (ABJ), climate data	Predict dengue cases based on climate factors	Dengue
Ningrum et al. (52)	AI, ML (Extra Trees, CatBoost), LSTM	Spatiotemporal, meteorological, surveillance data (Semarang City)	Develop AI spatiotemporal dengue prediction model	Dengue
Mazhar et al. (69)	Review (data-driven ML models)	Review data (surveillance, climate, epi)	Overview data-driven ML for dengue prediction	Dengue
Go (48)	ML (RF, LR, SVM, KNN)	Provincial morbidity data (Philippines)	Predict disease occurrences using ML	Multiple communicable diseases (HFMD, Dengue, Typhoid, Flu, etc.)
Flores et al. (32)	Analysis (focus on NLP algorithms)	Social media data	Explore algorithmic biases in NLP surveillance	–
Macintyre et al. (12)	Review (focus on AI-based EWS like EPIWATCH, HealthMap)	Review data (open source data)	Summarize AI potential in epidemic intelligence	Epidemic diseases
Gairola and Kumar (49)	ML (CNN features + classifiers: DT, KNN, NB, LR, RF, SVM), DL (CNNs: AlexNet, GoogleNet, VGG16), fusion	RGB image dataset (open source)	Develop ML method for image-based diagnosis	Monkeypox
Wang et al. (53)	DL (MSRD based on RNN), SVM, Lasso, Bayesian	Hospital reported case data, weather data	Develop MSRD model for fine-grained hospital EWS	Multiple IDs (HFMD, Influenza)
Roster et al. (27)	ML (RF, GB, FNN, SVR)	Epidemiological & meteorological data (Brazil)	Develop model to forecast monthly dengue cases	Dengue
Arslan and Benke (10)	AI, Data science	Online search queries, social media posts	Discuss AI/telehealth potential for early warning	Epidemics (COVID-19 context)
Apolinario-Arzube et al. (60)	DL, infodemiology	social networks, public reports, citizen input	Present collaborative infodemiology platform	Zika, dengue, chikungunya, influenza
Guo et al. (42)	AI (ANN—RTRL, EKF)	Notifiable disease case data (China)	Establish ANN model for early warning signals	Respiratory & Digestive IDs
Peterson (31)	–	–	Discuss ML/predictive analytics in clinical practice	Clinical outcomes (general)
Li et al. (46)	ML (logistic, Naive Bayesian, SVM), SVM-RFE	Patient prognosis data (hypertension cohort)	Establish risk early warning model	Cardiovascular diseases (stroke, heart/renal failure)

### 3.1 The growing need for early warning systems

The history of global health clearly shows the damaging impact of pandemics and epidemics, which have caused significant loss of life and lasting social and economic disruption (1, 2). Events such as the 1918 influenza pandemic, the 2003 Severe Acute Respiratory Syndrome (SARS) outbreak, and the recent COVID-19 pandemic serve as important reminders of the serious threat posed by infectious diseases (2, 8). These experiences show the importance of learning from the past and continuously improving our ability to detect and respond to outbreaks more effectively, informed by reviews of early warning system (EWS) effectiveness and global development experiences (7, 22, 23).

In addition to these historical lessons, several current global trends are increasing both the risk and speed of infectious disease outbreaks (1, 24). The rising number of zoonotic spillovers (diseases jumping from animals to humans), driven by factors such as deforestation and habitat loss, contributes to the emergence of new diseases, demanding integrated One Health approaches (2, 4). At the same time, the growing challenge of antimicrobial resistance is making the treatment of common bacterial infections more difficult, a problem that AI is being used to address (5, 25).

Globalization, driven by international travel and trade, enables pathogens to cross borders rapidly, increasing the potential for worldwide spread (1, 2). Urbanization leads to higher population densities, creating environments where diseases can spread more easily (1, 24). Furthermore, climate change is changing disease patterns and migration routes by expanding the habitats of disease vectors such as mosquitoes, making prediction and control more difficult and driving the need for climate-informed early warning systems (6, 26, 27). Together, these factors create a more complex and unstable environment for disease emergence and spread (1, 24).

In this changing situation, early warning systems (EWS) for epidemics are essential tools for preventing the rapid spread of infectious diseases and reducing their impact on public health (4, 12). These systems act as proactive defenses, allowing faster and more targeted responses to protect communities and save lives (16, 23). The ability to detect and understand outbreaks in their early stages is important for implementing timely interventions, such as quarantine measures, vaccination campaigns, and public education efforts, which can significantly change the course of an outbreak and lessen its overall burden (2, 13).

Early warnings offer a valuable window of opportunity to control an outbreak before it overwhelms healthcare systems and spreads further (10, 28). This emphasizes the importance of rapid, informed decision-making based on accurate and timely data–a challenge that modern technologies, particularly artificial intelligence, aim to address (29, 30).

### 3.2 How AI powers early warning systems

Addressing the challenges of modern disease surveillance requires tools capable of handling large and varied information; artificial intelligence (AI) offers such capabilities (1, 5). AI has become a powerful tool for processing and analyzing large datasets from diverse sources for infectious disease surveillance, operating at scales far beyond human capacity (16, 19, 31). It can analyze information from sources such as medical records, social media posts, news reports, and environmental monitoring devices (9, 10, 32). By analyzing these large volumes of varied data, AI applications in public health offer a more complete and timely understanding of disease dynamics (8, 33, 34).

AI detects early warning signals of infectious disease outbreaks through several mechanisms. It can identify anomalies–deviations from expected patterns–that may signal emerging public health threats (18, 19). AI algorithms are also capable of finding patterns in data that suggest the onset of a disease outbreak, allowing faster recognition of potential threats (1, 17, 35). For example, AI might detect an unusual spike in online searches for specific symptoms combined with increased social media posts about illness in a particular city, potentially indicating an outbreak days before official case counts rise (10, 12). Machine learning models are essential for finding correlations within large datasets that may indicate emerging outbreaks, enabling timely interventions (17, 36, 37).

Furthermore, AI is used in predictive modeling. AI-driven predictive analytics have played an important role in monitoring epidemiological trends, enabling public health officials to better anticipate and respond to potential outbreaks (16, 17, 35, 36). By creating predictive models, AI improves efforts in contact tracing and surveillance, helping to understand and control the spread of infectious diseases (11, 18, 38). Using historical data, environmental factors, and real-time surveillance information, machine learning models can forecast the spread and impact of infectious diseases with increasing accuracy (14, 27, 30, 39), enabling proactive resource allocation and more targeted public health measures (40).

The integration of AI into early warning systems significantly improves the speed and efficiency of outbreak detection and prediction compared to traditional methods (12, 38). By rapidly processing large amounts of data, AI can identify potential outbreaks much faster than conventional systems relying on manual data collection and analysis (1, 10, 41). This increased speed and efficiency support more timely and effective public health responses (9, 42).

However, it is important to recognize that AI serves as a valuable tool that supports and enhances, rather than replaces, traditional epidemiological methods and public health infrastructure (8, 12, 43). AI systems work alongside human-led efforts, providing new insights that help health professionals make better-informed decisions during outbreaks (31, 43, 44). The specific computational techniques that enable these functions are explored in the following section.

### 3.3 Artificial intelligence techniques and methodologies employed in early warning systems

Addressing the first review question, this section details the primary artificial intelligence techniques and methodologies employed in early warning systems for infectious disease surveillance, based on the reviewed literature. For readers interested in more detailed descriptions of these techniques, including their core principles and typical applications in the context of infectious disease EWS, please refer to Appendix A.

#### 3.3.1 Natural language processing (NLP)

Natural language processing (NLP) is important to analyze unstructured text data to detect early signals of infectious disease outbreaks (17, 32). AI systems use NLP to process large amounts of open-source data, including news reports and social media posts, to identify early warning signs of potential epidemics (12). NLP techniques can analyze user-generated content, detecting mentions of symptoms, self-reported illnesses, and concerns about disease spread in specific geographic areas, thus providing valuable real-time intelligence (19). Moreover, NLP has been applied within tools like medical chatbots (45) and to electronic medical record data to identify and characterize a broad range of symptoms associated with infectious diseases, improving the detail and speed of surveillance compared to structured data alone. By extracting relevant information from large amounts of online textual data, NLP enhances early outbreak detection, often identifying signals before official health notifications are released.

#### 3.3.2 Machine learning (ML)

Machine learning (ML) algorithms are widely used for pattern recognition, classification, and prediction in infectious disease surveillance (25, 37). These algorithms analyze structured and unstructured data from various sources to detect early warning signals of outbreaks (30). A variety of ML techniques are applied in epidemic and pandemic early warning systems. Among the most frequently employed are classification algorithms such as Support Vector Machines (SVM) and tree-based methods including Decision Trees (46–48), along with instance-based models like K-Nearest Neighbor (KNN) (37, 48), linear models like Logistic Regression (46, 48), and probabilistic classifiers such as Naive Bayes (46, 49). Several studies have shown that comparing or combining multiple ML techniques often improves the prediction of infectious disease incidence and trends, demonstrating their potential for forecasting disease dynamics (37, 48). Ensemble methods, a powerful ML extension, are discussed further in Section 3.3.5.

These algorithms are categorized into supervised, unsupervised, and reinforcement learning, serving distinct roles in early warning systems (EWS) for infectious diseases.

**Supervised learning** is the most commonly applied paradigm in the reviewed literature. These algorithms train on labeled data, where each instance is associated with a known outcome, enabling the model to predict outcomes for new, unseen inputs. In infectious disease surveillance, supervised learning is widely used to predict the likelihood or timing of future outbreaks based on epidemiological histories, climate patterns, and population mobility data; to classify cases or regions into predefined risk categories (e.g., high-risk vs. low-risk); and to support disease diagnosis using symptomatic data or medical imagery. Techniques such as SVM, Decision Trees, Logistic Regression, and Naive Bayes are frequently used within this framework.

**Unsupervised learning**, by contrast, works on unlabeled data and seeks to discover hidden structures, anomalies, or groupings without predefined outcomes. In EWS applications, it is commonly employed for anomaly detection (e.g., identifying unexpected spikes in symptom-related social media activity), clustering cases or outbreaks to uncover transmission dynamics, and topic modeling of text data to detect emerging public health concerns or novel symptom profiles.

**Reinforcement learning (RL)** involves an agent that interacts with an environment to learn optimal decision strategies through trial and error, aiming to maximize a cumulative reward over time. Although RL is less frequently applied in operational EWS compared to the other methods, it holds considerable potential. Notable applications include optimizing public health interventions, such as determining when and where to deploy vaccines or allocate resources, and developing adaptive control policies that respond quickly to evolving surveillance data. These applications, however, are more complex to implement and remain largely at the exploratory stage.

These learning paradigms provide an adaptable and growing toolkit for enhancing infectious disease EWS across a range of predictive and decision-support tasks.

#### 3.3.3 Deep learning (DL)

Deep learning (DL) techniques, a subset of ML utilizing neural networks, are increasingly recognized for their ability to handle complex, high-dimensional data in surveillance tasks (11, 41). Common architectures include Recurrent Neural Networks (RNNs), particularly Long Short-Term Memory (LSTM) networks, and Transformer models like Bidirectional Encoder Representations from Transformers (BERT) (50). DL models have shown great success in improving diagnostic accuracy and forecasting outbreaks by analyzing large datasets and recognizing complex patterns (3, 51). LSTM networks are especially well-suited for modeling time-based disease trends because they can retain information over long sequences (39, 52, 53). Transformer models like BERT are used for the classification and prioritization of textual information, such as news articles or social media posts, allowing faster and more efficient identification of outbreak-relevant data (12, 50). Other DL architectures found in the reviewed literature include Convolutional Neural Networks (CNNs), often applied to image data but also used in prediction models (49, 54), and custom networks like the Self-Excitation Attention Residual Network (SEAR) designed for influenza surveillance (13).

#### 3.3.4 Time series analysis

Time series analysis methods are frequently used to predict disease incidence and trends based on historical data patterns (37, 55). Statistical approaches, such as the Auto-Regressive Integrated Moving Average (ARIMA) model and its seasonal variants (SARIMA), consider trends, seasonality, and random fluctuations in time series data (39). These models are useful for tasks like predicting seasonal flu peaks or modeling reported case counts over time. Deep learning methods, particularly LSTMs (discussed in Section 3.3.3), are also commonly applied to time series forecasting in this domain (30, 52), alongside other neural network approaches (42).

#### 3.3.5 Ensemble learning

Ensemble learning techniques aim to improve the accuracy, robustness, and reliability of predictions by combining the outputs of multiple individual models (18). Common ensemble methods include Random Forest, which aggregates predictions from multiple decision trees (40, 56, 57), and various Boosting algorithms (such as Gradient Boosting, XGBoost, CatBoost, and LightGBM) that build models sequentially, with each new model correcting errors made by previous ones (13, 52, 56, 57). Another powerful ensemble technique is Stacking, where predictions from several different base models (e.g., ARIMA, SVM, LSTM) are used as inputs for a higher-level meta-learner to produce the final output (39). These ensemble methods often outperform single models by reducing variance and bias, leading to more reliable predictions for complex tasks like outbreak risk forecasting.

#### 3.3.6 Hybrid models

Finally, hybrid models that integrate different AI techniques or combine AI with statistical methods or domain-specific models are increasingly being explored to enhance early warning systems (9). By combining methods, hybrid approaches aim to leverage the different strengths of each component–balancing the interpretability of statistical models with the predictive power of deep learning algorithms or integrating physical models with machine learning (58). Examples include combining CNNs with Structural Equation Models (SEM) (54), using stacking ensembles as described above (39), or integrating Internet of Things (IoT) data streams with AI analysis platforms (59). These approaches create more powerful and flexible systems for infectious disease surveillance and prediction.

### 3.4 Data sources for AI-driven surveillance

To answer the second research question concerning data utilization, this section outlines the different data sources mainly used by these AI-driven systems. As shown in Table 3, common data sources for AI-powered infectious disease surveillance include news reports, social media platforms, electronic health records, environmental monitoring data, and official health notifications.

**Table 3:** Common data sources for AI-powered infectious disease surveillance.

**Data Source**	**Description**	**Utility in EWS**
News Reports	Online articles from various media outlets	Early detection of unusual health events, identification of potential outbreaks
Social Media	Posts and trends on platforms like Twitter, Facebook	Real-time public sentiment and discussion about symptoms and illnesses, early signals of outbreaks
Official Health Notifications	Reports from WHO, CDC, and other public health agencies	Confirmed case data, epidemiological trends, official alerts and guidance
Search Engine Queries	Aggregated search patterns for health-related terms	Indicator of public health concerns and potential increases in illness prevalence
Mobile Health & Wearable Data	Physiological data (temperature, heart rate, etc.) from devices	Early detection of individual and population-level health changes, potential early warning signs of infection
Environmental & Climatic Data	Temperature, precipitation, humidity, air quality, etc.	Understanding environmental factors influencing disease transmission, predicting suitable conditions for vector-borne diseases
Travel Data & Mobility Patterns	Airline passenger data, mobile phone location data	Tracking the movement of people and potential spread of diseases across regions
Genomic Data	Genetic sequences of pathogens	Identification of specific pathogens, tracking viral mutations, understanding pathogen evolution
Wastewater Surveillance	Analysis of sewage water for the presence of pathogens and their genetic material	Early detection of pathogens in a community, providing an unbiased measure of infection levels, including asymptomatic cases

#### 3.4.1 Digital and publicly available data

Open-source internet data provides a rich source of early outbreak signals, including news reports, social media activity, blogs, and health forums (12, 60). The internet serves as an extensive, real-time repository where public concerns, discussions about symptoms, and early reports of illness can emerge before official health notifications (10). Analyzing trends in social media posts [e.g., from platforms like Twitter (32, 50)], internet searches for health-related terms [e.g., using Baidu Index or Google Trends (61)], and online news articles can offer leading indicators of disease outbreaks.

#### 3.4.2 Health system and personal health data

Traditional epidemiological data from official health notifications and reports issued by organizations such as the World Health Organization (WHO) and national health agencies (e.g., the Centers for Disease Control and Prevention, CDC) are important to train and validate AI models (48). This includes specific datasets such as influenza-like illness (ILI) reports (13, 61), mandatory case reporting for diseases like dengue (27, 52), and hospital records, including electronic health records (EHR), laboratory information systems (LIS), and picture archiving and communication systems (PACS) (46, 62). Additionally, broader public health surveillance system data is frequently used (40). The emerging role of mobile health (mHealth) technologies and wearable device data offers a continuous stream of physiological indicators suitable for surveillance (29, 34, 63), although practical applications are still developing (17, 64).

#### 3.4.3 Environmental and contextual data

Environmental and climatic data, including temperature, humidity, and precipitation patterns, are important factors influencing the transmission of many infectious diseases, particularly vector-borne illnesses (6, 26, 27, 53). Travel data and human mobility patterns provide valuable insights into tracking and predicting the geographical spread of infectious diseases across regions (14, 15). Genomic data also plays an important role in identifying specific pathogens, tracking their evolution, and understanding potential changes in their characteristics (5, 8, 24). Finally, wastewater surveillance has emerged as a novel and unbiased data source for monitoring infection levels at the community level, capturing even asymptomatic cases (2, 22, 58).

#### 3.4.4 Integration of data sources

The integration of different data sources empowers AI-powered systems to achieve a more comprehensive and timely understanding of infectious disease threats (9). The ability to combine and analyze these varied datasets, often referred to as multi-source or multi-channel surveillance (7), is an important strength of AI in this domain (18, 38). Combining different data streams often provides a more robust and earlier signal than any single source alone (57), creating a synergistic effect for outbreak detection and prediction.

However, integrating such diverse datasets presents significant challenges, notably data heterogeneity, where information from various origins (e.g., structured climate data, unstructured social media text, epidemiological case counts) differs in format, scale, temporality, and reliability. Addressing these challenges is important for the effective application of AI in EWS.

For instance, in the surveillance of respiratory illnesses like influenza or COVID-19, many studies attempt to combine meteorological data (e.g., temperature, humidity) with indicators derived from web sources such as social media posts (for symptom mentions or public sentiment) or search query trends, alongside official epidemiological reports (18, 30, 50).

Successfully harmonizing these disparate data types typically involves several key steps.

##### 3.4.4.1 Temporal alignment

Datasets are often collected at different frequencies. A common approach is to aggregate them into consistent time units, such as daily or weekly summaries. For example, daily climate readings might be aligned with weekly aggregated social media sentiment scores and official case counts.

##### 3.4.4.2 Spatial aggregation

Information needs to be linked to common geographical units (e.g., city, county, or specific health districts). This might involve averaging climate data over a region or linking geolocated social media posts to defined administrative boundaries.

##### 3.4.4.3 Feature engineering

Raw data often requires transformation into formats suitable for AI model input. For social media, this could involve using natural language processing (NLP) to extract sentiment scores, topic frequencies, or mentions of specific symptoms. Climate variables might be used directly or transformed into anomaly indices (e.g., deviations from seasonal norms).

##### 3.4.4.4 Normalization and scaling

To prevent features with larger numerical values from disproportionately influencing model training, numerical data from different sources (e.g., temperature values ranging from −10 to 40°C and sentiment scores from −1 to 1) are typically normalized or scaled to a common range (e.g., 0 to 1 or *z*-scores).

Beyond harmonization, addressing the heterogeneity of combined data often relies on robust preprocessing pipelines to handle missing values and outliers, and the strategic selection of AI models. For example, ensemble methods like Random Forests have shown efficacy in managing complex datasets with a mix of structured (e.g., climate data) and unstructured (e.g., text-derived features) data (30). Furthermore, multimodal deep learning architectures are increasingly being explored for their capacity to learn joint representations from different data modalities simultaneously, offering a sophisticated approach to leveraging heterogeneous information for improved prediction accuracy in EWS.

### 3.5 Benefits of AI in infectious disease surveillance

This section addresses the third research question by summarizing the main reported benefits of applying artificial intelligence (AI) in infectious disease surveillance, based on the reviewed literature.

One of the key advantages of using AI in this field is its ability to enable earlier and faster detection of outbreaks compared to traditional surveillance systems (10, 12, 38). As noted in Section 3.1, speed is critical for effective response, and AI can reduce the delays associated with conventional methods by identifying epidemic signals much earlier (1, 23). A famous example is the BlueDot platform, which detected early signs of the COVID-19 outbreak before official reports were released (12).

This speed advantage is partly due to AI's ability to efficiently process and analyze large volumes of diverse data relevant to public health surveillance, as discussed in Section 3.4 (1, 16, 31). AI systems can handle data from sources such as medical records, laboratory results, social media, and environmental sensors (14, 18), extracting meaningful insights from information that would be too large or complex for human analysts to manage.

By processing this wide range of data using the techniques described in Section 3.3, AI can also improve the accuracy and precision of outbreak prediction and forecasting (13, 53). This leads to better-informed public health decision-making. Predictive analytics powered by AI have been important for monitoring epidemiological trends, allowing more accurate anticipation and faster responses to potential outbreaks (27, 30, 39). Studies show that AI and machine learning (ML) models often achieve higher performance metrics, such as accuracy, sensitivity, and lower error rates, compared to baseline or single-model approaches (40, 48, 52, 54).

Improved predictions also help optimize resource allocation and strengthen pandemic preparedness (6, 14). AI tools can analyze population health data to predict disease risk and spread, guiding the efficient distribution of resources such as hospital beds, medical supplies, and healthcare workers to areas of greatest need (15, 36). Timely and accurate predictions allow public health authorities to implement proactive measures, identify high-risk regions, and reduce the impact of outbreaks (27, 37, 48).

Furthermore, AI shows potential to address challenges in resource-constrained settings. In low-income countries, where human resources for traditional surveillance are limited, AI can automate processes and offer cost-effective solutions (12, 27). Technologies such as Edge AI can enable local analysis where centralized infrastructure is unavailable (63), and AI-powered point-of-care diagnostics can improve access to timely information (59). AI may also help overcome issues like data censorship by identifying signals from alternative sources, offering a more objective view of disease activity. However, the effectiveness of early warning systems can vary significantly between high- and low-resource settings (23).

### 3.6 Limitations and challenges of AI in infectious disease surveillance

Addressing the fourth review question, this section discusses the key limitations, challenges, and ethical considerations identified in the literature regarding the use of artificial intelligence (AI) for infectious disease surveillance.

#### 3.6.1 Data quality and biases

Despite its advantages, the use of AI in this field has important limitations. One major concern is the quality, completeness, and consistency of the data used to train and operate AI models (9, 63). Inaccurate, fragmented, or missing data can lead to unreliable outputs and poor model performance (19). Moreover, biases present in the data–such as underrepresentation of certain demographic or linguistic groups or lack of properly encoded information–can result in AI models that perform poorly for those groups, potentially worsening health inequities (11, 14, 32). Ensuring that datasets are comprehensive and representative is therefore critical to avoid biased outcomes (5, 17). Some models may also fail validation when applied to new datasets, showing issues with generalizability and potential overfitting (31, 51).

#### 3.6.2 Lack of transparency and understandability (“black box” problem)

Another critical issue is the lack of transparency in many advanced AI models, particularly deep learning algorithms, often referred to as the “black box” problem (9, 41). These systems often generate results without a clear explanation of how conclusions were reached (25, 65). This lack of understandability makes it difficult for public health professionals and clinicians to confirm, trust, or troubleshoot model outputs (31). Ongoing research in explainable AI (XAI) aims to address this challenge (41, 44, 56). Without understanding the reasoning behind AI predictions, it becomes difficult to correct errors or explain decisions based on them.

#### 3.6.3 The necessity of human expertise and oversight

While AI tools can process and analyze data at scale, human oversight remains essential for interpreting results and making appropriate public health decisions (14, 43). AI systems are best used as complementary tools alongside traditional epidemiological methods, rather than as replacements (8, 12). Human expertise is necessary to validate findings, evaluate anomalies, contextualize AI outputs, and ensure that insights are appropriately applied in complex real-world situations (1, 31).

#### 3.6.4 Challenges in integrating AI into existing infrastructure

Integrating AI into existing public health infrastructures presents significant technical and organizational challenges (1, 44). These include issues with the interoperability of different systems, where varying data formats and protocols restrict the seamless exchange of information needed for comprehensive AI analysis (19, 63). Other challenges involve inconsistent data-sharing protocols (7), the need for robust local data infrastructure (36), and limited workforce training or expertise to utilize AI tools effectively (1). Maintaining data security and confidentiality while ensuring data availability for real-time processing is also a significant operational concern (63). Difficulties in enabling distributed, collaborative decision-making across different platforms or institutions have also been noted (28).

#### 3.6.5 Ethical considerations

The use of AI in infectious disease surveillance raises numerous ethical challenges (15, 16, 65). Data privacy and security are top concerns, given the sensitivity of personal health information (8, 11). This requires robust governance frameworks and privacy-preserving techniques (14). Technologies such as federated learning, which allow model training on decentralized data without sharing raw information, are being explored to mitigate these risks (29, 63). Questions remain regarding who controls health data, how consent is obtained [especially when using public data sources like social media (50)], and how to ensure responsible data use (11).

Additionally, AI systems may continue or even worsen existing social inequities when trained on biased data, resulting in unfair treatment or exclusion of disadvantaged groups (25, 32, 44). The issue of accountability is also important: when AI systems support public health decisions, it is important to define who is responsible, particularly when errors occur (11). Finally, ensuring equitable access to AI technologies and their benefits for all populations is essential to avoid large global health disparities (36, 44).

### 3.7 Existing and proposed AI-based early warning systems for infectious diseases

Despite the challenges mentioned in the previous section, several AI-based early warning systems have been developed and deployed, demonstrating the practical application of these technologies (38, 55). These systems vary in their approaches, data sources, and specific techniques employed. To better understand the operational characteristics of some of the AI-enhanced EWS, Table 4 provides a comparative summary of the three main systems (i.e., HealthMap, BlueDot, and EPIWATCH) showing their input data, AI approaches, output features, and aspects related to latency.

**Table 4:** Comparison of selected AI-based early warning systems.

**Feature**	**HealthMap (73)**	**BlueDot (12)**	**EPIWATCH (66)**
Primary input data	News media (Google News, etc.), official reports (WHO, ProMED-mail), web sources (blogs), social media, user eyewitness reports	Official health notifications (WHO), news articles, animal/plant disease networks, travel data (airline ticketing), remote sensing data, client-provided government data	Curated sources (WHO, CDC, Outbreak News Today), non-curated (Google News with >4,000 search terms), social media (Twitter, future)
AI techniques used	Automated classification (Fisher-Robinson Bayesian filtering), NLP (text processing algorithm for identifying, classifying, mapping)	AI (NLP in 65 languages), human moderation, transport network modeling, clustering tools for hotspot identification	AI-based event filtering, NLP (BERT for article classification & prioritization with 88.2% accuracy for relevance), named entity recognition, human review
Key output features	Geographic mapping of events, linked reports, alerts by disease/syndrome, timelines, mobile app (“Outbreaks Near Me”)	Alerts to clients, hotspot identification, risk analysis (details often proprietary)	Public dashboard with searchable/sortable outbreak reports, GIS mapping, analytics, risk analysis tools (EPIRISK, FLUCAST, ORIGINS)
Reported latency	Hourly data collection. Detected COVID-19 signals on Dec 30, 2019 (1 day before official Chinese acknowledgment)	Near real-time analysis. Identified undiagnosed pneumonia (COVID-19) on day of WHO declaration (Dec 31, 2019)	Curated sources: near real-time; Non-curated: daily. Detected COVID-19 signals on day of WHO announcement
System-specific	Reports all health events, not specific to epidemics. Accuracy of automated categorization reported as 84% in earlier literature	Commercial system; specific accuracy metrics for outbreak prediction not publicly detailed in the comparative review	Reported 88.2% accuracy for AI (BERT) in assessing article relevance to outbreaks. Focus on infectious diseases and syndromes

#### 3.7.1 Systems primarily using web and public data sources

Several systems focus on using open-source internet data for early detection of signals. HealthMap is a fully automated system that monitors health events, including infectious diseases, by using natural language processing (NLP) to analyze real-time data from web sources such as news reports and health forums, providing a global view (12). EPIWATCH is another AI-based system that generates automated early warnings by analyzing open-source data with techniques like NLP and named entity recognition (NER) (12). It has been used, for example, to study the effects of conflicts on disease epidemiology and to track respiratory illness trends (66).

Epitweetr, developed by the European Center for Disease Prevention and Control (ECDC), specifically monitors tweets related to infectious diseases, allowing filtering by location and time (12). An earlier example, Google Flu Trends, attempted to predict influenza prevalence using search query data (12). While pioneering, it faced challenges related to accuracy, including matching noise instead of true signals (overfitting) and seasonal biases. Other research continues to explore the utility of social media platforms like Twitter (50) and search engine query data (10, 61) for surveillance.

#### 3.7.2 Systems integrating diverse data sources

Other systems aim to integrate a wider variety of data sources to improve predictions and address limitations such as reporting delays. BlueDot, a well-known Canadian platform, gained attention for its early detection of the COVID-19 outbreak (12). It uses AI to analyze diverse global data, including airline ticketing data and official reports, demonstrating how combining non-traditional sources can potentially overcome delays or censorship in official reporting.

The Global Biosurveillance Portal (GBSP) is a web-based system that integrates data from multiple web applications and government sources for timely responses, using AI-based predictive analysis (12). The Metabiota Epidemic Tracker uses big data analytics and cloud computing to simulate epidemic events and conduct risk analysis across numerous pathogens (12).

Beyond these named platforms, many studies describe frameworks or models that integrate multiple data streams. These include systems that combine: (1) community-level or hospital surveillance data (such as influenza-like illness reports, case notifications, and electronic health records) with external factors like weather or web data (4, 40, 53, 61, 62); (2) vector surveillance data with climate parameters and case data, especially for diseases like dengue (26, 27, 52); (3) food safety surveillance data with human case data for foodborne illnesses (57); (4) data from networked point-of-care testing devices using Internet of Things (IoT) technology (59); (5) wastewater-based epidemiology data with hydraulic modeling (58); and (6) inputs from social networks, public reports, and direct citizen participation (60).

Many proposed systems emphasize multi-source, multi-channel, or multi-point trigger approaches to improve sensitivity and robustness (7).

These examples show the diverse approaches and data sources used by existing and proposed AI-powered early warning systems. While some systems show significant promise, ongoing development and refinement are important for improving their accuracy, reliability, and acceptance by public health authorities (9, 22, 67, 68).

### 3.8 AI applications in specific infectious diseases

The practical application and impact of artificial intelligence (AI) in early warning and disease management have been demonstrated across several major infectious disease outbreaks and surveillance efforts, as illustrated by the following examples.

#### 3.8.1 COVID-19

During the recent pandemic, AI played several important roles (8, 41). Platforms like BlueDot provided early detection of the outbreak, demonstrating the benefits discussed in previous sections (12). AI-supported radiology tools aided diagnosis through the automated analysis of medical images such as CT scans (51). Social media and search query data were analyzed using AI to track the virus's spread and monitor public sentiment (33). AI models also predicted COVID-19 spread based on mobility data and other factors (1). Furthermore, tools like EPIWATCH analyzed the impact of global events on disease epidemiology (66), and other AI models were developed using COVID-19 case data to improve forecasting and understand transmission patterns (11, 39, 61).

#### 3.8.2 Influenza

Influenza surveillance has also benefited significantly from AI applications. Google Flu Trends represented an early attempt to predict influenza activity using search queries, illustrating both the potential and limitations related to data quality and accuracy, as discussed in previous sections (12). More recently, tools like EPIWATCH have included components for predicting flu season severity (66). Deep learning models, including custom attention-based networks, have been developed specifically for influenza surveillance using influenza-like illness (ILI) data, demonstrating strong early warning performance in some settings (13, 61). AI-driven analysis of search queries and other data sources continues to be explored for forecasting influenza trends (30, 42).

#### 3.8.3 Ebola

During the 2014 West Africa outbreak, AI algorithms were applied to analyze large datasets to track virus spread and predict potential hotspots, reportedly aiding more efficient resource allocation and containment efforts. Platforms such as HealthMap, BlueDot, and Metabiota included Ebola in their monitoring activities (12). While detailed examples specific to Ebola were less prominent in the reviewed literature compared to COVID-19 or influenza, the event shows the potential for predictive models in outbreak response.

#### 3.8.4 Dengue fever

Given its significant global burden, dengue fever is another area where AI and machine learning (ML) models are actively being developed and applied. Research focuses on forecasting dengue cases or outbreaks using epidemiological surveillance data combined with climate or meteorological variables (26, 27, 69). AI approaches, including spatiotemporal models, are being designed specifically for dengue early warning systems (52). Systems may incorporate vector surveillance data alongside case and climate information (26), or integrate data from platforms involving citizen participation (60). The goal is to provide timely predictions to support public health interventions and vector control efforts (6, 48).

#### 3.8.5 Other infectious diseases

AI and ML techniques are also being applied to a growing range of other infectious diseases beyond the major examples above. In response to the monkeypox outbreaks, researchers have developed machine learning models for diagnosis, using either clinical symptom data–sometimes incorporating explainable AI (XAI) techniques to improve trust and transparency (56)–or analyzing image data of lesions to aid detection (49).

Applications in tuberculosis (TB) surveillance include using machine learning to predict the risk of relapse in patients and exploring the transferability of predictive models trained on other respiratory illnesses, such as COVID-19, to forecast TB case numbers (25, 39, 54). For foodborne illnesses, tree-based machine learning algorithms have been applied to integrated food safety surveillance data and human case reports to predict the spatiotemporal patterns of salmonellosis outbreaks (57).

Nosocomial (hospital-acquired) infections represent another area where machine learning methods are used on hospital surveillance data, incorporating factors such as antibiotic use and operational metrics to assess risks and predict infection incidence (40). Furthermore, AI models are being developed for broader categories, such as classifying various vector-borne diseases based on symptomatology (47) or using neural networks for early warning across multiple notifiable respiratory and digestive tract diseases (42, 48).

Specific applications also include surveillance systems for outbreaks like Lassa fever (55) and predicting trends for diseases such as hand, foot, and mouth disease (53).

### 3.9 Future trends and advancements in AI for early detection of infectious disease outbreaks

Finally, addressing the fifth research question, this section explores the emerging trends and future directions for the development and application of artificial intelligence (AI) in this field, as suggested by the reviewed literature.

#### 3.9.1 Data integration and algorithm improvement

Future developments will involve enhanced integration of diverse data sources, including real-time streams from social media, wearable devices, environmental sensors, wastewater monitoring, and genomic sequencing (16, 50, 59). There is an increasing emphasis on combining multi-sectoral data under frameworks such as One Health (4), and integrating clinical information with external factors like climate patterns or web searches (22, 53).

At the same time, AI models will continue to evolve, with the development of more advanced and accurate algorithms. This includes further refinement of deep learning models and ensemble techniques, aimed at improving predictive capabilities (5, 38, 46). These advancements are expected to enhance the reliability of AI systems and address challenges related to data quality and completeness (9). Continuous model updating and optimization through iterative feedback and expert judgment will be essential for maintaining model relevance and performance (13).

#### 3.9.2 Enhanced transparency and privacy

Addressing ethical concerns will remain important. A growing focus on explainable AI (XAI) is anticipated to improve transparency and trust in AI-driven systems (25, 41). XAI directly tackles the “black box” challenge discussed in Section 3.6.2, allowing stakeholders to better understand the reasoning behind AI predictions (5, 50, 56).

Several specific XAI techniques are gaining importance for their utility in interpreting complex models, thereby enhancing practical relevance. For example, **Local Interpretable Model-agnostic Explanations (LIME)** offers a method to explain the predictions of any machine learning model by approximating its behavior with a simpler, interpretable model (e.g., a linear model) locally around a specific instance being predicted (70). In the EWS context, LIME could thus help public health officials understand why an otherwise opaque AI system flagged a particular region or time point as high-risk for an outbreak.

Another widely adopted technique is **SHapley Additive exPlanations (SHAP)**, which utilizes a game theory approach, specifically Shapley values, to assign an importance value to each feature for a particular prediction (71). This value indicates the feature's contribution to the model's output, allowing for a quantitative assessment of how different data inputs (e.g., specific symptoms reported on social media, recent mobility patterns, or prevailing climate conditions) contributed to an AI model's forecast of increased disease incidence.

Furthermore, particularly for deep learning models such as Transformers and some Convolutional Neural Networks (CNNs), **Attention Visualization** provides valuable insights by allowing for the inspection of internal attention mechanisms. These visualizations can reveal which parts of the input data the model focused on most when making a decision (72). For instance, in an EWS processing news articles or social media posts, attention weights could identify specific keywords or phrases that triggered an alert; similarly, in time-series forecasting, such visualizations could identify which historical data points were most influential for a given prediction.

The adoption and further development of these and other XAI methods are crucial for building trust and facilitating the practical deployment of AI in critical public health decision-making processes. Ongoing research continues to refine these techniques and develop new ones tailored to the complexities of AI in healthcare and infectious disease surveillance.

In parallel, emerging privacy-enhancing technologies such as federated learning (29, 63), which allows AI models to be trained on decentralized data without sharing raw sensitive information, will be equally important.

The promise of federated learning (FL) in the EWS context primarily derives from its ability to train robust AI models collaboratively across multiple entities (e.g., hospitals, clinics, individual wearable devices) without the need to centralize sensitive raw patient data, thus directly addressing critical data privacy and security concerns (29, 63). The reviewed literature describes conceptual **architectures** for FL in health surveillance. For instance, Javed et al. introduce a framework leveraging FL with data from wearable health monitoring “gages” for the early diagnosis of infectious diseases like COVID-19, dengue, and tuberculosis, emphasizing its potential for lower power consumption on distributed devices (29). Tian et al. (28) propose an FL-based “alliance monitoring” module specifically for medical institutions as part of a broader blockchain-enabled EWS, facilitating secure inter-institutional data sharing and collaborative model building. These architectures typically involve local model training on decentralized datasets, with only aggregated parameters or model updates being shared, often via a central server (though serverless peer-to-peer models are also conceived), to build a more generalized global model.

Regarding **technical feasibility**, while FL offers significant advantages for privacy and access to diverse data, its practical implementation in EWS is subject to several considerations identified in the literature. Benefits include the potential for more accurate and generalizable models from varied data sources (29) and reduced latency when combined with edge computing (63). However, significant challenges persist, including managing statistical heterogeneity (non-IID data) across different participating nodes, the communication overhead required for transmitting model updates, ensuring the security and privacy of the model updates themselves against inference attacks, the computational demands on local devices or institutions, and the complexities of system interoperability and integration into existing public health infrastructures (63).

While comprehensive **case studies** detailing the large-scale deployment and empirical performance of FL-based EWS for specific infectious diseases were still emerging within our review period (2018-onward), the reviewed literature strongly advocates for its potential and outlines numerous proposed applications. Beyond specific disease mentions by Javed et al. (29), the general applicability of FL is highlighted for scenarios requiring collaborative analysis of distributed health data while preserving privacy, which is fundamental for effective and equitable EWS. Overcoming the identified technical and logistical hurdles is crucial for transitioning FL from a promising concept to a widely adopted, impactful technology in routine public health surveillance for infectious diseases.

Blockchain technology is also being explored for secure data sharing and smart contract applications (22, 28). These technologies are expected to support collaborative AI development while safeguarding data privacy and security.

#### 3.9.3 Broadening scope: novel threats and personalization

Advancements are anticipated in the real-time monitoring and early detection of novel and emerging infectious diseases (EIDs) (7, 8, 24). AI systems will increasingly focus on detecting unusual symptom clusters through syndromic surveillance, potentially identifying new or unexpected infectious threats before their causes are fully understood (23).

At the same time, the application of AI in personalized and precision public health is expected to expand (3, 31). Future strategies could involve customizing warnings or preventive advice based on individual risk profiles derived from data from wearable devices, genomic information, or particular clinical factors (17, 43, 44).

#### 3.9.4 Global collaboration and standardization

Increased global collaboration and data sharing will be essential to enhance pandemic preparedness (16, 25). Developing standardized AI tools and data protocols will facilitate more effective global disease surveillance and response (8), helping to overcome integration challenges and reducing data biases described in previous sections.

Establishing cross-sectoral partnerships among public health agencies, healthcare providers, academic institutions, and technology developers will be critical for sharing expertise, co-developing solutions, and fostering innovation (5, 19, 32).

## 4 Limitations of this review

This systematic review, while aiming to provide a comprehensive overview of the recent landscape of AI applications in EWS for infectious diseases, is subject to several methodological limitations that should be considered when interpreting its findings.

Firstly, the scope of our literature retrieval was primarily based on the Semantic Scholar database. Although Semantic Scholar is an extensive, updated, AI-driven platform indexing an extensive number of academic papers, the dependence on a single primary database, despite our structured search strategy, may mean that some relevant studies indexed exclusively in other databases (e.g., Web of Science, Scopus, PubMed Central for specific biomedical aspects) might have been missed. This could potentially introduce a degree of selection bias.

Secondly, our review included a language restriction, focusing only on studies published in English. This was a practical decision to ensure consistent interpretation and data, but it inevitably excludes research published in other languages. Therefore, valuable insights and AI applications developed or reported in non-English literature, particularly from regions where English is not the primary language of scientific publication, may not be represented in our synthesis, potentially skewing the geographical representation of research activities.

Thirdly, as with most systematic reviews, there is a potential for publication bias. Studies reporting positive, novel, or statistically significant findings are often more likely to be published than those with null, negative, or inconclusive results. This could lead to an overrepresentation of successful AI applications or an underestimation of the challenges and failures in the field of AI for EWS.

Fourthly, the timeframe for our search (2018 onwards) was chosen to focus on recent advancements in this rapidly growing field. While this provides a contemporary overview, it means that foundational or earlier relevant studies published before 2018 were not included in this specific review.

Finally, the significant heterogeneity observed across the 67 included studies in terms of AI methodologies, specific diseases, datasets, and evaluation metrics made it challenging to conduct a direct quantitative comparison or meta-analysis of the performance of different AI approaches. Our review, therefore, primarily provides a qualitative synthesis and mapping of the reported landscape.

## 5 Discussion

Historical pandemics and contemporary factors such as globalization, climate change, and zoonotic spillover (diseases transmitted from animals to humans) show the urgent need to enhance global preparedness against infectious diseases (1, 2, 4, 6). In response, this systematic review evaluated the current state of the use of AI in early warning systems (EWS) for infectious disease surveillance, summarizing findings from 67 relevant studies. Specifically, it addressed five research questions related to: primary AI methods, data sources, perceived benefits, significant challenges, and future trends. One consideration is that our review was based on literature retrieved from Semantic Scholar. Although this database covers a broad spectrum of scientific publications, it may not include all relevant studies indexed in other sources such as Web of Science or Scopus. However, given its integration of diverse publication sources and strong coverage of peer-reviewed literature, we believe this approach was appropriate for the scope and objectives of our review. Overall, the findings suggest that AI has the potential to transform infectious disease surveillance from reactive approaches into proactive, data-driven predictions, although several technical, practical, and ethical barriers still limit its general implementation.

Regarding the first research question on primary AI methods, the reviewed studies show a clear shift from traditional statistical methods toward more advanced machine learning (ML) and deep learning (DL) techniques. ML classifiers, such as support vector machines (SVM), logistic regression, and k-nearest neighbors (KNN), remain popular for disease prediction and classification tasks (46, 48). Ensemble methods, particularly Random Forests, consistently achieve strong performance in predicting hospital-acquired infections (40), forecasting communicable diseases (48), and identifying foodborne illness outbreaks (57). Additionally, DL models that capture temporal patterns, such as Long Short-Term Memory (LSTM) networks, have proven particularly effective for forecasting diseases like influenza and Dengue fever (52, 53). Innovations in customized DL architectures, demonstrated by attention-based SEAR networks for influenza surveillance (13), further illustrate the evolution of the field. Meanwhile, natural language processing (NLP) techniques have become important for extracting insights from unstructured text in news articles, social media, and clinical reports, enabling real-time tracking of public sentiment and symptom reporting (10, 12, 32). Hybrid approaches, combining multiple algorithms or integrating AI with traditional epidemiological models, are increasingly adopted to improve overall predictive accuracy and system robustness (39, 58).

In addressing the second research question about data sources, modern AI-driven EWS emphasize the integration of diverse and large-scale datasets. While traditional epidemiological sources–such as case reports, influenza-like illness (ILI) counts, and hospital records–remain important (13, 62), the full potential of AI appears through combining these traditional sources with non-traditional datasets. Web-based data, including news articles, social media platforms [e.g., Twitter (50)], and search engine queries [e.g., Baidu Index (61)], offer real-time indicators of emerging health concerns. Environmental and climatic datasets are important for forecasting vector-borne illnesses like Dengue fever (26, 27), as well as other weather-sensitive diseases (6). Emerging data sources, such as wastewater surveillance, provide unbiased, community-level indicators of disease activity (22, 58), and genomic sequencing enables precise identification and tracking of pathogens (8, 24). Mobile health technologies and wearable devices offer future potential for personalized health monitoring, although their integration into broader public health surveillance remains limited at this time (29, 34, 63). The integration of such diverse data enhances predictive accuracy but also introduces substantial challenges related to data quality, consistency, and interoperability (9, 63).

Furthermore, a significant overarching challenge implicitly linked to data quality, model transparency, and ethical considerations is the generalizability of AI models, particularly in the context of cross-region or cross-population applications. Our review notes that issues with model generalizability and the risk of poor performance when applied to new, distinct datasets are recognized limitations in the field (as discussed in Section 3.6.1). While the concept of transfer learning between different diseases was noted in some reviewed literature (Section 3.8.5), a deep, specific exploration into the methodologies, comparative effectiveness, and challenges of cross-region transfer learning (e.g., adapting models developed in one continent for robust application in another with different demographic, environmental, or healthcare system characteristics) was beyond the defined scope of our primary research questions. Our review aimed to provide a broad assessment of the current landscape of AI techniques, data sources, reported benefits, and broadly identified challenges within EWS for infectious diseases. The complexities of developing, validating, and implementing effective and equitable cross-region transfer learning strategies represent a substantial and critical research domain in their own right.

Regarding the third research question (benefits), AI-based early warning systems primarily enhance the time and accuracy of outbreak detection. Multiple studies and real-world systems, such as BlueDot's early identification of COVID-19 (12), illustrate how AI can detect outbreaks sooner than traditional surveillance methods (10, 38), thus enabling faster and more effective public health interventions (23). Improved predictive accuracy further supports health authorities in allocating resources and responding effectively to outbreaks (14, 27, 36, 48, 52, 54). Additionally, AI-driven automation of data processing may offer cost savings, particularly in resource-limited settings (12), although equitable access to these advanced technologies remains an important concern (23).

Despite these clear benefits, the fourth research question identifies substantial limitations. The quality, completeness, and representativeness of input data determine AI performance; thus, poor data quality inevitably leads to unreliable predictions (“garbage in, garbage out”) (9). Biases inherent in data collection processes–such as underreporting or limited digital access–can result in biased AI outputs that intensify existing health inequities (11, 14, 32). The “black box” nature of complex DL models, characterized by their lack of transparency, also represents a significant barrier to clinician and public health official adoption (31, 41). While Explainable AI (XAI) methods are emerging to address this challenge, they remain underdeveloped (5, 56). Additional challenges include technical difficulties in integrating AI systems into existing public health infrastructure, along with complex ethical considerations around privacy, consent, fairness, accountability, and potential misuse of data (11, 14, 19, 32, 50, 63). Finally, human expertise continues to be essential for interpreting AI-generated insights and making important public health decisions (8, 43).

Considering future trends (fifth research question), the field is moving toward integrating diverse datasets, developing more sophisticated, transparent algorithms, and adopting privacy-preserving technologies such as federated learning and blockchain (3, 5, 16, 28, 29, 41). However, achieving these goals will require global collaboration, standardized data practices, sustained investment in infrastructure and workforce training, and clear ethical frameworks to guide responsible AI development and deployment (1, 8, 14, 19).

## 6 Conclusion

This systematic review shows the growing importance and rapid development of artificial intelligence (AI) in early warning systems for infectious diseases. AI methods have the potential to greatly improve the speed, accuracy, and effectiveness of outbreak detection and prediction. By analyzing large and varied data sources, ranging from traditional health records to digital media, environmental measurements, and wastewater surveillance, AI can provide earlier and more precise warnings. This advantage has been clearly demonstrated for diseases such as COVID-19, influenza, and Dengue fever.

However, significant challenges remain, preventing AI from being widely implemented. Issues related to data quality, missing or biased data, and transparency in complex AI (“black box”) models must be carefully addressed. The need to explain how AI reaches its conclusions (“explainable AI”) is necessary to build trust among healthcare professionals and public health authorities. Additionally, there are technical difficulties in combining and managing large datasets, and ethical concerns about privacy, fairness, and accountability. It is also important to ensure that AI systems support human decision-making rather than replace it.

While AI offers great promise for improving infectious disease surveillance and global health preparedness, achieving these benefits requires a coordinated effort. Continued investment in developing transparent, fair, and ethical AI technologies is needed, along with improvements in data management, training of health workers, and international cooperation.

## Data Availability

The original contributions presented in the study are included in the article/Supplementary material, further inquiries can be directed to the corresponding author.
