# Supplementary material for: Artificial intelligence in early warning systems for infectious disease surveillance: a systematic review
Source: Front Public Health. 2025 Jun 23;13:1609615. doi: 10.3389/fpubh.2025.1609615 (PMC12230060; doi:10.3389/fpubh.2025.1609615)
Supplement: Supplementary file 1 [file Data_Sheet_1.pdf]

A Appendix A: Summaries of Key Artificial Intelligence Techniques and Methodologies

This appendix provides brief summaries of the core principles and typical applications in Early Warning Systems (EWS) for infectious diseases for the primary Artificial Intelligence (AI) techniques and methodologies discussed in Section 3.3 of this review.

1. Natural Language Processing (NLP)

Core Principle: A field of AI focused on enabling computers to derive meaning from, interpret, and generate human language (text and speech). It involves techniques like tokenization, part-of-speech tagging, named entity recognition, sentiment analysis, topic modeling, and machine translation [1].

Application in EWS: Analyzing unstructured textual data from diverse sources such as news reports, social media posts, scientific publications, electronic health records (EHRs), and medical chatbot interactions to detect early signals of outbreaks, track public sentiment and concern, identify mentions of symptoms, and extract relevant epidemiological information.

2. Machine Learning (ML) - General

Core Principle: A branch of AI where systems learn from data to identify patterns, make decisions, and improve their performance over time without being explicitly programmed for each specific task. ML algorithms can be broadly categorized into supervised learning (learning from labeled data), unsupervised learning (finding patterns in unlabeled data), and reinforcement learning (learning through trial and error with rewards/penalties) [2].

Application in EWS: Used for a wide range of tasks including classification (e.g., identifying high-risk individuals or regions), regression (e.g., predicting disease incidence), clustering (e.g., grouping similar cases or outbreaks), and pattern recognition in epidemiological, environmental, and behavioral data to detect anomalies or predict future trends.

2.1. Support Vector Machines (SVM)

Core Principle: A supervised learning algorithm primarily used for classification tasks, though adaptable for regression (Support Vector Regression - SVR). SVMs aim to find an optimal hyperplane in a high-dimensional feature space that distinctly classifies data points by maximizing the margin between the classes. Kernel functions can be used to map data to higher dimensions, allowing for non-linear separation [3].

Application in EWS: Classifying disease states (e.g., infected vs. non-infected), predicting outbreak risk levels, and identifying patterns in complex datasets.

2.2. Decision Trees

Core Principle: A supervised learning algorithm that creates a tree-like model of decisions and their possible consequences. Each internal node represents a "test" on an attribute (e.g., a symptom), each branch represents the outcome of the test, and each leaf node represents a class label (e.g., disease presence) or a continuous value (in regression trees) [4].

Application in EWS: Classifying cases based on symptoms or risk factors, identifying key predictive factors for disease transmission, and providing interpretable models for decision support.

2.3. K-Nearest Neighbor (KNN)

Core Principle: A non-parametric, instance-based supervised learning algorithm used for both classification and regression. It classifies a new data point based on the majority class (for classification) or average value (for regression) of its 'k' closest neighbors in the feature space, typically measured by a distance metric like Euclidean distance [5].

Application in EWS: Classifying new potential cases based on their similarity to known cases, identifying spatial or temporal clusters of disease, and anomaly detection.

2.4. Logistic Regression

Core Principle: A statistical supervised learning algorithm used for binary or multiclass classification. It models the probability of a categorical dependent variable (e.g., outbreak occurrence, infection status) using a logistic (sigmoid) function applied to a linear combination of independent variables [6].

Application in EWS: Predicting the probability of an outbreak, assessing the risk of infection based on various factors, and modeling binary outcomes in epidemiological studies.

2.5. Naive Bayes

Core Principle: A probabilistic supervised learning classifier based on Bayes' theorem, with a "naive" assumption of conditional independence between features given the class. Despite this simplification, it often performs well, especially in text classification [7].

Application in EWS: Classifying text data from news or social media for relevance to outbreaks or sentiment, predicting disease likelihood based on a set of symptoms, and spam filtering in communication channels.

3. Deep Learning (DL) - General

Core Principle: A subfield of ML based on artificial neural networks with multiple layers (hence "deep") that learn hierarchical representations of data. These networks automatically discover complex structures in large datasets by transforming input data through successive layers of non-linear processing units [8].

Application in EWS: Handling complex, high-dimensional data for tasks like medical image analysis (e.g., X-rays, CT scans), advanced time series forecasting, sophisticated natural language understanding from large text corpora, and genomic data analysis.

3.1. Recurrent Neural Networks (RNNs)

Core Principle: A type of neural network designed to process sequential data (e.g., time series, text) by having connections that form directed cycles. This allows them to maintain an internal state or "memory" that captures information about previous inputs in the sequence, influencing the processing of current and future inputs [9].

Application in EWS: Modeling time-based disease trends, analyzing sequences in text (e.g., symptom progression descriptions) or genomic data, and forecasting future values in time series.

3.2. Long Short-Term Memory (LSTM) Networks

Core Principle: A specialized type of Recurrent Neural Network (RNN) architecture designed to learn long-range dependencies in sequential data. LSTMs employ gating mechanisms (input, forget, and output gates) that regulate the flow of information into and out of a memory cell. This allows them to selectively retain relevant information and discard irrelevant data over extended time periods, effectively addressing the vanishing gradient problem that can hinder learning in traditional RNNs [10].

Application in EWS: Widely used for forecasting disease incidence and trends over time (e.g., for influenza, COVID-19, Dengue fever), analyzing patterns in time-ordered epidemiological data, and processing sequential information from diverse sources like social media or electronic health records for early warning signals.

3.3. Transformer Models

Core Principle: A deep learning architecture that relies heavily on the "self-attention" mechanism, which allows the model to weigh the importance of different parts of the input sequence (e.g., words in a sentence) when processing it, capturing contextual relationships effectively. Unlike RNNs, Transformers can process entire sequences in parallel. BERT (Bidirectional Encoder Representations from Transformers) is a prominent pre-trained Transformer model for NLP tasks [11].

Application in EWS: Advanced NLP tasks such as classifying and prioritizing textual information (news articles, social media posts, scientific literature) for outbreak relevance, understanding context in medical texts, question answering, and information extraction.

3.4. Convolutional Neural Networks (CNNs)

Core Principle: A type of deep learning network particularly effective for processing grid-like data, such as images (2D grids of pixels) or audio (1D grids of samples). CNNs use convolutional layers with learnable filters (kernels) to automatically detect spatial hierarchies of features, from low-level edges and textures to high-level objects or patterns. Other key components include pooling layers (for down-sampling) and fully connected layers (for classification) [12].

Application in EWS: Analyzing medical images (e.g., CT scans for pneumonia detection in COVID-19, skin lesion images for monkeypox), and can also be adapted for sequence data or time series analysis in some predictive models.

3.5. Self-Excitation Attention Residual Network (SEAR)

Core Principle: The Self-Excitation Attention Residual Network (SEAR) is a deep learning model developed to improve early warning systems for influenza outbreaks in China. It is specifically designed to handle regional variations in epidemic patterns by using a combination of attention mechanisms and residual learning. The model takes daily estimates of the reproduction number (Rt) as input and predicts whether the value exceeds a predefined warning threshold, indicating a potential outbreak.

SEAR uses a four-path architecture that integrates squeeze-and-excitation (SE) attention modules and residual blocks to enhance feature extraction. This structure allows the model to focus on the most relevant epidemic signals while ignoring less important data. After processing through different paths, the features are concatenated and passed through a sigmoid layer to generate binary warning predictions.

Compared to traditional models like logistic regression, SVM, random forest, XGBoost, and LSTM, SEAR achieved higher performance across multiple metrics, including accuracy, recall, F1 score, and ROC AUC. Its flexible design

enables it to adapt to feedback and new data, making it a robust and generalizable tool for real-time infectious disease surveillance [13].

Application in EWS: Primarily for forecasting infectious disease trends, such as influenza, by learning from historical epidemiological data and potentially other influencing factors.

4. Time Series Analysis - General

Core Principle: Involves statistical methods and ML/DL techniques used to analyze time-ordered data points (sequences taken at successive, equally spaced points in time). The goal is to extract meaningful statistics, identify patterns (like trends, seasonality, cyclicity, and autocorrelation), and make forecasts [14].

Application in EWS: Predicting future disease incidence and trends based on historical patterns (e.g., weekly influenza case counts, daily COVID-19 hospitalizations), detecting anomalies in surveillance data, and understanding the temporal dynamics of outbreaks.

4.1. Auto-Regressive Integrated Moving Average (ARIMA) / SARIMA

Core Principle: ARIMA is a class of statistical models for analyzing and forecasting time series data. It combines three components: AR (Auto-Regressive), where the model depends on previous values of the time series; I (Integrated), which involves differencing the raw observations to make the time series stationary (i.e., its statistical properties do not change over time); and MA (Moving Average), where the model incorporates the dependency between an observation and a residual error from a moving average model applied to lagged observations. SARIMA (Seasonal ARIMA) extends ARIMA to handle time series with a seasonal component [15, 16].

Application in EWS: Forecasting incidence of seasonal diseases (e.g., influenza) or other infectious diseases with temporal patterns, modeling reported case counts over time.

5. Ensemble Learning

Core Principle: ML techniques that strategically combine predictions from multiple individual models (base learners) to produce a single, often more accurate, robust, and reliable prediction than any individual model. The idea is that multiple diverse models can collectively make better decisions by compensating for each other's weaknesses [17].

Application in EWS: Improving the accuracy and reliability of predictions for complex tasks like outbreak risk forecasting, disease incidence prediction, and diagnostic support systems.

5.1. Random Forest

Core Principle: An ensemble learning method that constructs a multitude of decision trees at training time. For classification, the output is the class selected by most trees; for regression, it is the average of the outputs of individual trees. It uses bagging (bootstrap aggregating) by training each tree on a random subset of the data and also considers only a random subset of features for splitting at each node, which helps to reduce variance and prevent overfitting [18].

Application in EWS: Predicting disease outbreaks, forecasting communicable disease trends, identifying important features for disease transmission, and risk assessment for nosocomial infections or foodborne illnesses.

5.2. Boosting (e.g., Gradient Boosting, XGBoost, CatBoost, LightGBM)

Core Principle: A family of ensemble algorithms that build models sequentially, where each new model (a weak learner, typically a decision tree) attempts to correct the errors made by its predecessors. Models are added iteratively, with each new learner focusing more on the instances that previous learners misclassified or predicted with larger errors. Gradient Boosting is a common framework; XGBoost, CatBoost, and LightGBM are highly optimized and popular implementations offering improvements in speed, performance, regularization, and handling of categorical data [19].

Application in EWS: Achieving high-accuracy predictions for disease outbreaks, forecasting incidence, and risk modeling in complex epidemiological datasets.

5.3. Stacking Approaches for ML

Core Principle: An ensemble learning technique that combines multiple different types of classification or regression models (base models or level-0 models) by training a meta-learner (level-1 model). The base models are trained on the full training set, and their predictions are then used as input features to train the meta-learner, which produces the final output [20].

Application in EWS: Improving overall predictive accuracy by leveraging the diverse strengths of different modeling approaches (e.g., combining ARIMA, SVM, and LSTM predictions for a final forecast).

6. Hybrid Models

Core Principle: Approaches that integrate different AI techniques (e.g., combining NLP with ML classifiers, or DL with traditional ML) or combine AI

methods with conventional statistical models, mechanistic epidemiological models (e.g., SIR models), or domain-specific physical models. The aim is to leverage the complementary strengths of each component to create more powerful, flexible, accurate, or interpretable systems [21].

Application in EWS: Developing more robust and comprehensive surveillance and prediction systems, such as integrating IoT data streams with AI analysis platforms, combining CNNs with Structural Equation Models, or using outputs from mechanistic models as inputs for ML models.

References

[1] O'Connor J, McDermott I. Principles of NLP: What it is, how it works. Singing Dragon; 2013.

[2] Alpaydin E. Introduction to machine learning. MIT press; 2020.

[3] Noble WS. What is a support vector machine? Nature biotechnology. 2006;24(12):1565-7.

[4] Kingsford C, Salzberg SL. What are decision trees? Nature biotechnology. 2008;26(9):1011-3.

[5] Fix E, Hodges JL. Discriminatory Analysis, Nonparametric Discrimination: Consistency Properties. Randolph Field, Texas: USAF School of Aviation Medicine; 1951. 4.

[6] Berkson J. The Application of the Logit Transformation to the Analysis of Dosage-Effect Curves. Biometrics. 1944;1(1):1-13.

[7] Zhang H, Li D. Naïve Bayes text classifier. In: 2007 IEEE international conference on granular computing (GRC 2007). IEEE; 2007. p. 708-8.

[8] Bengio Y, Goodfellow I, Courville A, et al. Deep learning. vol. 1. MIT press Cambridge, MA, USA; 2017.

[9] Anderson JA. An introduction to neural networks. MIT press; 1995.

[10] Yu Y, Si X, Hu C, Zhang J. A review of recurrent neural networks: LSTM cells and network architectures. Neural computation. 2019;31(7):1235-70.

[11] Tunstall L, Von Werra L, Wolf T. Natural language processing with transformers. O'Reilly Media, Inc.; 2022.

[12] Gonzalez RC. Deep convolutional neural networks [lecture notes]. IEEE Signal Processing Magazine. 2018;35(6):79-87.

[13] Yang L, Yang J, He Y, Zhang M, Han X, Hu X, et al. Enhancing infectious diseases early warning: A deep learning approach for influenza surveillance in China. Preventive Medicine Reports. 2024;43.

[14] Hamilton JD. Time series analysis. Princeton university press; 2020.

[15] Shumway RH, Stoffer DS, Shumway RH, Stoffer DS. ARIMA models. Time series analysis and its applications: with R examples. 2017:75-163.

[16] Dubey AK, Kumar A, García-Díaz V, Sharma AK, Kanhaiya K. Study and analysis of SARIMA and LSTM in forecasting time series data. Sustainable Energy Technologies and Assessments. 2021;47:101474.

[17] Polikar R. Ensemble learning. Ensemble machine learning: Methods and applications. 2012:1-34.

[18] Breiman L. Random forests. Machine learning. 2001;45:5-32.

[19] Bentéjac C, Csörgő A, Martínez-Muñoz G. A comparative analysis of gradient boosting algorithms. Artificial Intelligence Review. 2021;54:1937-67.

[20] Pavlyshenko B. Using stacking approaches for machine learning models. In: 2018 IEEE second international conference on data stream mining & processing (DSMP). IEEE; 2018. p. 255-8.

[21] Azevedo BF, Rocha AMA, Pereira AI. Hybrid approaches to optimization and machine learning methods: a systematic literature review. Machine Learning. 2024;113(7):4055-97.
